# Supplementary material for: Viral RNA Metagenomics of Hyalomma Ticks Collected from Dromedary Camels in Makkah Province, Saudi Arabia
Source: Viruses. 2021 Jul 18;13(7):1396. doi: 10.3390/v13071396 (PMC8310382; doi:10.3390/v13071396)
Supplement: Supplementary file 1 [file viruses-13-01396-s001.zip › viruses-1280647-supplementary.pdf]

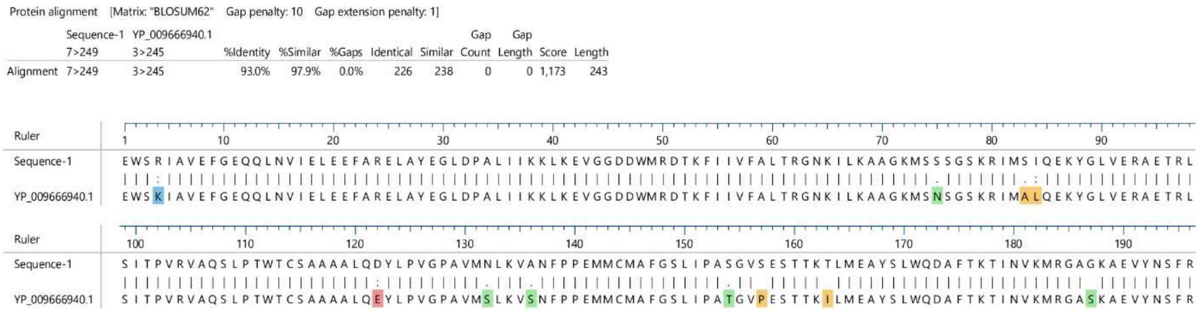

Figure S1. BLOSUM62 score showing substitutions between the newly identified Guertu virus in Saudi Arabia and the strain (YP_009666940.1) identified in China.

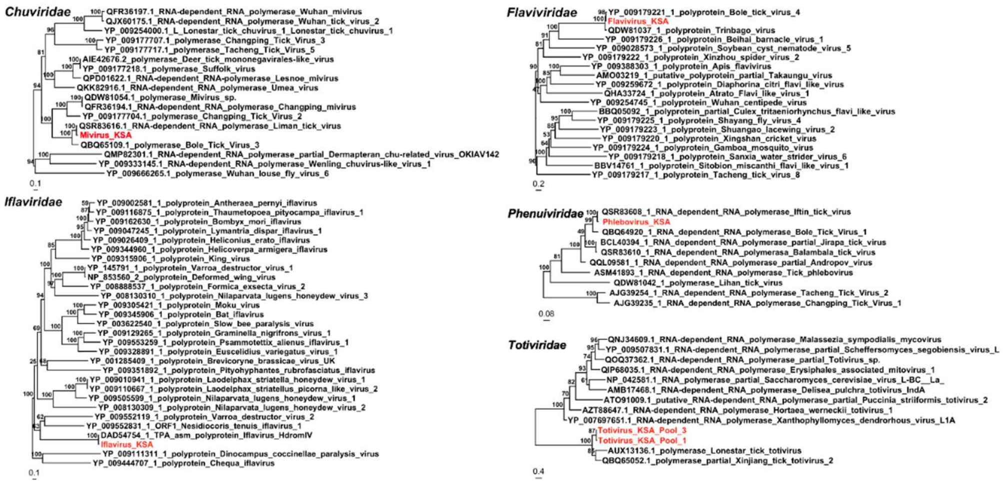

Figure S2. Phylogentic trees of viruses that have not been associated with human diseases. The viruses detected in Saudi Arabia are marked with red colour.
